# 3D Human Motion Editing and Synthesis: A Survey

**DOI:** 10.1155/2014/104535

**Published:** 2014-06-19

**Authors:** Xin Wang, Qiudi Chen, Wanliang Wang

**Affiliations:** ^1^College of Computer Science and Technology, Zhejiang University of Technology, Hangzhou 310023, China; ^2^Key Laboratory of Visual Media Intelligent Process Technology of Zhejiang Province, Hangzhou 310023, China

## Abstract

The ways to compute the kinematics and dynamic quantities of human bodies in motion have been studied in many biomedical papers. This paper presents a comprehensive survey of 3D human motion editing and synthesis techniques. Firstly, four types of methods for 3D human motion synthesis are introduced and compared. Secondly, motion capture data representation, motion editing, and motion synthesis are reviewed successively. Finally, future research directions are suggested.

## 1. Introduction

To obtain realistic 3D human motion data, artists, designers, and computer experts have proposed many methods. Although these methods have made a significant progress in 3D human motion capture technology, human motion data have a high degree of freedom (DOF). In addition, the human eye is sensitive to human motion distortion. Therefore, many difficulties and challenges in 3D human motion synthesis still exist. These proposed methods can be roughly divided into the following four categories: (1) manual methods, (2) physics-based methods, (3) video-based methods, and (4) motion capture data-driven methods. Among these four types, the motion capture data-driven methods have been extensively applied because of their realistic results and real-time data processing algorithms. This paper reviews and analyses the four types of methods and focuses on the typical technology of motion capture data-driven methods.

## 2. Classification of 3D Human Motion Synthesis

Manual methods refer not only to the steps of manually setting the DOFs of human joints in all key frames before generating continuous human motion through interpolation but also to the specialised algorithms which are used to synthesise specific motion [1, 2]. These algorithms are relatively simple and efficient. However, producing a new motion requires a new specialised algorithm each time. The resultant motion is less exquisite and realistic than the data from motion capture equipment.

The idea of physics-based methods [3–5] is based on real human movements in accordance with the physical law. As such, the mass distribution of each part of the human body can be obtained following the research methods of biomechanics. Then, ordinary differential equations (ODEs) are established based on the torque and the trajectory of each joint following Newton's law. Finally, the trajectory of each joint is obtained by solving the ODE, and the entire range of human motion is determined. The greatest difficulty for physics-based methods is designing a specific equation of motion. Even the equation generating a specific movement, which corresponds to the physical law, lacks details and has no individuality.

Video-based methods [6] use computer vision technology such as contour tracing and feature extraction to extract human motion features from videos taken from different angles. On the one hand, we can obtain the 3D motion information of each joint from these features and synthesize the 3D motion of the entire human body [7]. On the other hand, we can use these features to obtain the whole body 3D spatial posture in each frame. In the latter case, we generally do not consider the motion information of each joint. The 3D human motion data obtained can be divided into small segments that are then recombined to synthesize new motion [8]. Video-based methods are classified into two categories, namely, the top-down category and the bottom-up category.

Motion capture data-driven methods mainly refer to the reuse of existing 3D motion data to generate new motion. Human motion data mainly come from the original data captured by the motion capture equipment as well as from manual methods and physics-based methods; even the output of data-driven methods can serve as a source of human motion data. The methods for motion data reuse are as follows: (1) using the signal processing method to edit the motion data of individual joint and individual freedom at the lower level, (2) adjusting the emotion of a specific motion at the higher level, (3) connecting short segments to generate a long segment, (4) extracting some common motions from multiple motion segments, (5) recovering the motion information of each joint from several joints, and (6) modifying the motion data based on the physical law.

Table 1 shows the comparison of the advantages and disadvantages of the four methods. Fundamental differences can be observed among these methods in terms of their approach to problem solving. However, each method has its own advantages and disadvantages. As such, the hybrid usage of these methods, such as the mixture of motion capture data-driven methods and video-based methods [9] and the combination of motion capture data-driven methods and physics-based methods [10, 11], is applied in practical situations.

## 3. Motion Capture Data Representation

**Figure 1:**
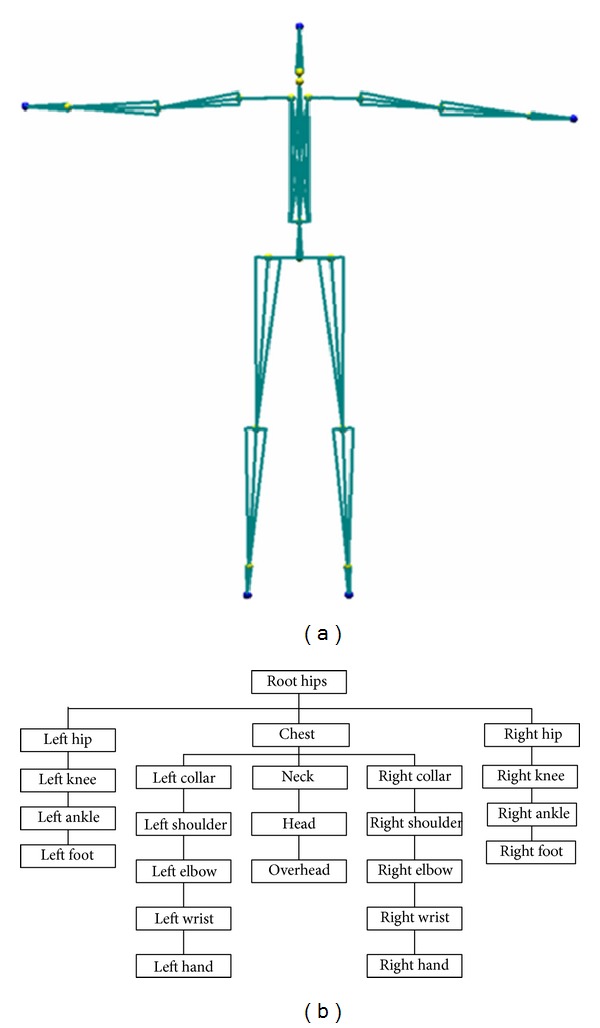
Human skeleton structure.

The storage format of motion capture data is different according to different manufacturers. In general, the skeleton structure shown in Figure 1(a) is used to indicate the human joint chain, with each joint connected based on the hierarchical structure shown in Figure 1(b).

The root in the skeleton structure records the offset of the human body in the world coordinate, whereas the other joints record their translation and rotation information with respect to their parent joint. In general, the translation of the child joint with respect to its parent is a fixed value because it represents the bone length between two joints. The spatial information of all joints can affect the spatial location of the joint in the sublayer. The root translation represents the movement of the whole skeleton. By contrast, the other joints only rotate. The translation vector is a 3D spatial vector, and its rotation can be represented by a rotation matrix, Euler angles, or quaternion. Human motion can be expressed by a discrete time vector function **m**(*t*) = [**p**(*t*), **q**_1_(*t*), **q**_2_(*t*),…, **q**_*n*_(*t*)]  (1 ≤ *t* ≤ *T*), where **p**(*t*) ∈ **R** ^3^ is the root translation information and **q**_*i*_(*t*)  (*i* = 1,2,…, *n*) is the *i*th joint rotation information.

Although the general concept of motion capture data is the translation and rotation of structured information, the original data captured by motion capture equipment should in fact undergo several stages of processing to obtain structured information [12–14].

In addition, some motion capture data include not only the motion data but also some constraints which express certain attributes, such as physical constraints (the foot must be above the ground plane) and features of the motion type (the number of times you clap your hands when you feel excited). These constraints can be considered as metadata and can be assigned to a single frame, a sequence, or the whole motion clip.

## 4. Motion Capture Data-Driven Methods

Motion capture equipment can generate realistic and smooth motion. However, the equipment is expensive, the motion capture process is laborious and time consuming, and the results do not meet the prerequirements. These drawbacks require the original data to be processed further. To address these issues, several researchers have proposed many motion editing methods which can be applied to captured motion data and other motion data obtained using other methods. These motion editing methods usually modify some attributes to satisfy particular demands in animation (meet user's specifications). However, the generated motion is a short segment similar to the original segment.

In recent years, researchers have also proposed the concept of motion synthesis to synthesise continuous, long-time, and constraint-conformed human motion data. Firstly, this motion synthesis technique is used to extract elements from a motion clip. Then, these elements are organised through a specific data structure (such as motion graphs [15] and Markov chain [16]). Finally, based on the user's requirements, appropriate elements are searched, and a new motion is synthesised. Motion synthesis is more flexible than motion editing because it can generate a variety of motions, thus significantly improving the utilisation of the original motion.

### 4.1. Motion Editing Methods

Motion capture equipment can record the performer's motion realistically. However, editing these data is difficult because of the following factors. (1) Large volume of data; to continuously record the performer's action, the sampling rate of the motion capture device should be high. Some optical devices can reach greater than 1,000 fps, leading to a large amount of data that is difficult to edit. (2) Lack of structured information; traditional computer animation controls the final generated animation by the key frames or the input parameters. However, we only obtain a small amount of original data by motion capturing, which cannot provide the motion feature. Furthermore, the ways to modify these data to affect motion effectively are vague. (3) Modifying some attributes may tend to change other attributes which should not be modified.

Motion editing methods thus focus on how to efficiently modify one attribute of the motion data in accordance with the requirements while keeping the other attributes unchanged. Existing motion editing methods can be classified based on the modified attribute (as shown in Table 2).

### 4.2. Motion Synthesis Methods

**Figure 2:**
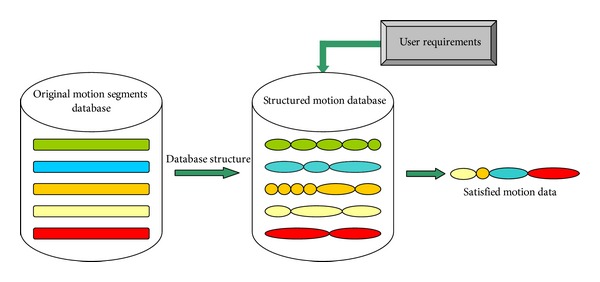
General process used in the motion synthesis method.

In the early part of 1996, researchers proposed motion synthesis by example [17], but the DOF was only 5. In recent years, motion synthesis methods have progressed to synthesize multiple DOFs (such as in Figure 1 more than 70) and fine motion. In general, synthesis methods involve outline processing, as shown in Figure 2. Firstly, the features of the original motion segments are analysed. Then, the feature between segments or of the single segment is used to build a motion database which is well designed and can provide user interface to express demand. The motion database also has the ability to connect, smoothen, enquire, and perform other motion editing operations to obtain the satisfactory motion data.

The present typical motion synthesis methods can be divided into two categories, namely, the motion graph-based category and the statistical model-based category. No absolute boundary exists between the two methods, as the method based on the motion graph may use the concept of statistics in a step; the same goes for the statistical model.

#### 4.2.1. Motion Synthesis Methods Based on Motion Graph

The graph-based motion synthesis method has been used earlier in the game industry [18]. The graph construction process is as follows: firstly, the designers design the basic motion clips. Then, the interactive software is used to connect these clips. Lastly, the original clips and the connected clips are connected through a manually designed graph structure [19]. In this way, the motion graph structure is satisfactory because it can obtain the required motion in real time through searching. In addition, the connection between these vertexes is simple and able to meet the demand for motion control of the game characters. In recent years, some researchers have proposed several methods for automatically constructing motion graphs. Some of these methods have been proposed by Kovar et al., Lee et al., and Arikan and Forsyth in 2002 [15, 16, 20].

The general idea of the three studies is the same, that is, finding a set of similarities between a group of motion data clips, then constructing a motion graph by constructing a transition clip between similarities, and finally searching the graph to obtain the satisfactory motion. The three studies differ in the following four aspects: (1) detection of similarity, (2) generation of the transitions, (3) graph construction method, and (4) goal-achieved graph search.

*(1) Detection of Similarity*. In this step, the problem to be solved is how to evaluate the similarity between any two frames to determine whether to add a transition clip between them.

The three studies all designed the evaluation formula of the similarity considering the joint position, velocity, acceleration, and other factors. In these evaluation formulas, researchers empirically set different weights corresponding to different joints based on the distribution of human motion sensitive areas, such as in Lee's study [16], where the weight of the shoulder, elbow, hip, knee, and chest is set to 1, whereas the weight of the neck, ankles, toes, and wrist is set to 0.

*(2) Generation of the Transitions*. In this step, the problem to be solved is how to generate a transition clip to smoothly join the motion before the *i*th frame and the motion after the *j*th frame if the addition of an edge between two frames has been determined.

Arikan and Forsyth did not generate a transition clip between the original motion clips but dealt with discontinuities using a form of localised smoothing [20] at each joint connection (often has first-order discontinuity) to obtain smooth motion signals.

Kovar et al. used linear interpolation. They created a transition from the *i*th frame of the first motion to the *j*th frame of the second motion by linearly interpolating the root positions, performing spherical linear interpolation on joint rotations, and placing additional constraints on the desired motion [15].

Jehee and Yong used the hierarchical motion fitting algorithm [21], established four cases based on the differences between the constraint interval relative to the transition clip interval, and then considered different constraint maintenance strategies to generate transitions based on different situations.

**Figure 3:**
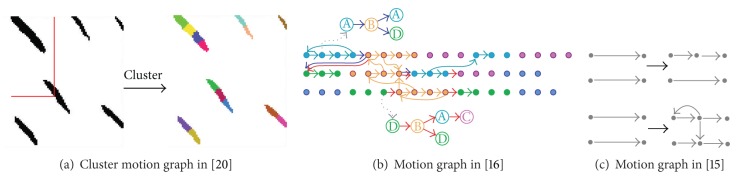
Three different motion graph-constructing methods.

*(3) Graph Construction Method*. Arikan and Forsyth represented the original clip as a node and then used an edge to connect two frames if the similarity function value exceeded a threshold. Given two consecutive frames in the original data with high similarity, the results of the similarity distribution are shown in Figure 3(a) [20]. That is, the edges in a cluster can be clustered to an edge, and a binary tree can also be used to present the connection of two clips by edge labels, thus constructing a hierarchical motion graph. This graph has the same nodes in each level, with two edges at the lower level connected to one edge at the higher level.

As suggested in the work of Kovar et al. [15], edges are used to present the motion clips, and nodes serve as choice points where these motions are joined seamlessly. Then, a node is inserted to divide an initial clip into two smaller clips. We can also insert a transition joining two nodes using motion blending to construct a motion graph (as shown in Figure 3(b)).

Lee et al. [16] presented a two-layer structure to represent human motion data. The lower layer retains the details of the original motion data, whereas the higher layer is a generalization of the motion data. The lower layer is a directed graph composed of nodes and edges. Each specific motion frame of the original motion is a node, and an edge must be placed between consecutive frames, as well as similarities. The higher layer is a statistical model constructing a data structure called cluster tree at each motion frame which generalizes a set of similar human actions. Each node in the higher layer is the root of the corresponding cluster tree (as shown in Figure 3(c)).

*(4) Graph Search Meets the Goal*. Arikan and Forsyth synthesized constrained motion sequences by searching appropriate paths in this graph using a randomized search method [20] which starts with a set of paths in the graph randomly, scores each path and all possible mutations, does every possible mutation, compares the satisfaction of the constraints to the original path, accepts the mutations that are better than the original paths, repeats until no better path can be generated through mutations, and obtains the final path.

Kovar et al. defined an objective function and then used branch and bound to find the optimal path as the final motion path in graph searching [15].

Lee et al. determined the cluster path *p* on the constructed cluster tree, evaluated the joint probability *P*(*s*, *p*) of these paths (where *s* is the sequence of motion frames), and finally selected the most probable path as the final path [16].

Based on these three studies, many other researchers further explored human motion synthesis based on motion graph. Gleicher et al. constructed a simple graph to facilitate efficient planning of character motions. A user-guided process manually selects the character poses, and the system automatically synthesizes the transitions connecting these poses [22]. Sung presented a novel continuous motion graph for crowd simulation. This motion graph can create motions with arbitrary trajectories and speed up the motion synthesizing time while satisfying constraints exactly [23]. Reitsma and Pollard used task-based metrics to evaluate the capability of a motion graph to create animations. They examined the capability of typical motion graphs across tasks and environments and evaluated the extent to which a motion graph will fulfill requirements [24]. Zhao and Safonova proposed a new method for building a well-connected motion graph with good connectivity and only smooth transitions. Firstly, the method builds similar interpolated motion clips and then constructs a motion graph and decreases its size [25]. Zhaoy et al. also proposed an automatic approach called iterative subgraph algorithm to select a good motion set [26]. Ren et al. studied the optimisation of motion graphs, including enhancing the connectivity, streamlining the size, and improving the natural transitions [27]. Zong et al. created an automatic motion graph with a high degree of polymerisation nodes which extract key postures by adopting dimension reduction and nonparametric density estimation analysis [28]. Liu et al. focused on the semantic control of motion graph-based motion synthesis. Relational features, a self-learning procedure and semantic control, are implemented, thus providing user with a high level of intuitive semantic controls [29]. Yu et al. proposed a path editing method based on motion graphs. They detected the motion clips by minimising the average frame distance between the blending frames and proposed Enhanced Dynamic Time Wrapping to solve the optimisation problem [30].

#### 4.2.2. The Statistical Motion Synthesis Model

The typical motion synthesis methods based on the statistical model are discussed below.

Mattew et al. considered style to be variations in mapping from qualitative states to quantitative observations and then constructed a generic human state machine combined with cross entropy optimisation, annealing, and other automatically learning methods which can also control the state machine using various settings and can generate motion in a variety of styles.

Tanco et al. presented a system that can generate transition between two arbitrary key frames. The states of Markov chain are built by clustering, and the original motion capture data serve as implicit states. The model comprises two levels. The first level can generate a coarse motion by traversing the states of the Markov chain. The second level relates the states of the Markov chain with segments of the original motions in the database and generates a realistic synthetic motion based on these segments. Matthew and Aaron and Tanco and Hilton [31, 32] used a two-level hidden Markov model (HMM) to present motion data.

Li et al. modelled the local dynamics (of a segment of frames) by using a linear dynamic system (LDS) and global dynamics (of the entire sequence) by switching between these linear systems [33]. Yan Li proposed a concept called motion texton which is represented by an LDS that captures the dynamics shared by all instances of this texton in the motion sequence. Yan Li also designed a maximum likelihood algorithm to learn the motion textons and their relationship from the captured dance motion. The learnt motion texture can then be used to generate new animations automatically and/or edit animation sequences interactively.

Hsu et al. learned to translate by analysing the differences between performances of the same content in terms of input and output styles. This method relies on a linear time-invariant (LTI) model to represent stylistic differences [34]. Once the model is estimated with system identification, our system is capable of translating streaming input with simple linear operations at each frame.

Pullen et al. proposed the synthesis of joint angle and translation data based on the information in motion capture data and divided training data into frequency bands using wavelet decomposition.

Correlations are modelled with a kernel-based representation of the joint probability distributions of the features. Lastly, the data are synthesised by sampling from these densities and improving the results using a new iterative maximisation technique [35]. This technique has been applied in the synthesis of the joint angle and translation data of a wallaby hopping on a treadmill and is useful for the animation of repetitive motions, such as walking or running with low DOF. The quality of the generated motion still needs further verification when extended to human motion with high DOFs.

Bowden extended the point distribution models (PDMs) of representation and recognition of deformation to human motion and joints state data variation based on time [36]. Then, human motion synthesis, detection, and identification from the learnt PDMs were conducted.

HMMs do not encode high-order temporal dependencies easily. Local optima are frequently encountered by iterative optimisation techniques when learning HMMs. Thus, model topology and size are often highly constrained prior to training. Galata et al. proposed the use of the variable-length Markov model as a simple [37] yet powerful and efficient mechanism for determining behavioural dependencies and long-term and short-term constraints. Although learnt behaviour models can be used to animate human activity, control over future behaviour is lost once the beginning motion is specified.

Jenkins and Matarić extended the Isomap algorithm to incorporate spatiotemporal structure [38] and then used dimension reduction to manually segment motion data and extract primitive motion modules (as verbs in [19]). Then, another iteration of spatiotemporal Isomap was performed to extract metalevel behaviour modules (as adverbs in [19]). The system can synthesise a stream of human motions from a user-selected metalevel behaviour. Motion synthesis based on behaviour was proposed in [1]. Jenkins and Matarić automatically derived vocabularies of motion modules from human motion data [38]. The limitation of the study is that users can only synthesise metalevel motion.

Wei et al. showed how statistical motion priors can be seamlessly combined with physical constraints for human motion modelling and generation. The key idea is to learn a nonlinear probabilistic force field function and combine it with the physical constraints in a probabilistic framework [39].

In addition to linear systems such as LDS and LTI, a nonlinear system has been used to model motion data. Wang et al. used the Gaussian process dynamical model (GPDM) for human motion modelling and synthesis of new continuous motions. GPDM is a kind of nonlinear hidden variable model suitable for temporal data. GPDM considers the temporal structure of the input data [40].

Overall, the motion synthesis methods presented in [35–37] are focused on intermediate body tracking and gesture recognition and not on realistic human motion. As such, synthetic motion tends to be rough.

#### 4.2.3. Other Motion Synthesis Methods Based on Motion Capture Data

Some other motion synthesis methods based on motion data, aside from motion graph and statistical model, are discussed in this section. Pullen and Bregler [41] allowed the animator to sketch an animation by setting a small number of key frames, segmenting these key frames into many monotonic curve segments, matching each curve segment with the presegmented motion database, and finally joining the optimal match in the library to produce the constraint-satisfied and rich-detailed motion.

Liu et al. used an optimisation algorithm to extract key frames from human motion capture data by combining the genetic algorithm and the probabilistic simplex method. This method provides the optimal number of key frames by using the genetic algorithm while accelerating the search speed through the simplex local search technology [42].

Jin et al. proposed a new method to automatically extract key frames from animation sequences. The method uses animation saliency computed on the original data and reconstructs the input animation. This method can be applied equally in skeletal and mesh animations [43].

Yujie et al. proposed a framework and algorithm for 3D human motion synthesis based on nonlinear manifold learning. In the framework, high-dimensional motion samples are mapped into low-dimensional manifold using the nonlinear dimensionality reduction method [44].

## 5. Discussion

3D human motion synthesis technology has made significant breakthroughs in the last decade. Although motion capture devices and data processing algorithms have improved, many problems still need to be solved, and new research directions must be explored.

*(1) Motion Database Organisation*. Although the motion synthesis technologies described previously have designed how human motion data can be stored structurally, the motion database structures formed with these methods are not always adequate and require tedious manual adjustments by the database designer to achieve a good structure. However, manual adjustments of the motion database can only guarantee the quality of the local motion data. Whether the type of motion data of the whole motion database is sufficient and whether the synthetic range of motion is large enough should be evaluated. These evaluation methods of the overall performance of the motion database still need further exploration.

The database [45] consists of a binary tree and node transition graphs. The human motion database [46] constitutes several components, namely, the cross-validation dataset, the generalisation dataset, the compositionality dataset, and the interaction dataset.

*(2) Motion Database Compression*. The main problem of motion data compression is how to decrease the storage capacity of motion data without decreasing the quality of the motion data. One intuitive idea is to extract key frames from the motion capture data and then recover the original motion data from these key frames. Many researchers have also proposed a number of methods [47–50], but the performance of these methods should be further improved.

*(3) Motion Database Retrieval*. Search methods of motion data can generally be divided into the following two categories. (1) Metadata-based search: this search method is relatively simple, and its running speed is fast. Nevertheless, the quality of the outcome depends on the original marked metadata. The time-consuming and subjective metadata annotation process limits the application of these search methods. (2) Similarity-based automated data search: the basis of this method is the existing function which can well define the similarity between media data. Given that the similarity between the relationships of motion data can be established based on the similarity function, the retrieval of motion data can be achieved. At present, the most commonly used method [51–54] is the similarity-based automated data search.

Numaguchi et al. developed a puppet interface system for the retrieval of motion capture data. They introduced a novel motion retrieval algorithm called the dual subspace projection method that outperforms conventional pose-based retrieval methods [55]. Chao et al. retrieved motion by drawing the motion strokes; this technique is more convenient than opening a motion file as the query example [56].

*(4) Motion Data Quality Evaluation*. Whether the motion data achieved by a variety of motion synthesis technologies are naturally integrated or concise (no redundancy and noise) is generally judged by the observation of the naked eyes. However, when the motion database is large or the motion data need to be used in a real-time virtual environment, manually determining the quality of motion data becomes difficult or even impossible. Some researchers have proposed automated motion data evaluation methods [57–60]. However, most of these methods are only applicable for a specific type of motion and have limited performance.

*(5) Group Motion Synthesis*. The general motion synthesis technology is mainly used for the synthesis of one individual. With regard to the synthesis of multiple characters, the task of motion synthesis undergoes a qualitative change from quantitative change. To control group motion, group behaviour, path planning, collision detection, and other issues must be considered. In recent years, group motion synthesis has become a hot research topic, and certain outcomes have been achieved [61–63].

van Toll et al. used crowd density information to guide a large number of characters by building a navigation mesh and weighing the desirability of routes based on the crowd density along the path [64].

*(6) New Ideas of Human Motion Synthesis*. Recently, many new ideas distinct from those of the previous four methods have been proposed. Park and Hodgins proposed the method of directly capturing skin deformation to reconstruct human motion [65]. To synthesise motion, Chai and Hodgins used low-dimensional control signals from a user's performance supplemented by a database of prerecorded human motions [66].

*(7) Reactive Human Motion Synthesis*. The main problem of reactive human motion synthesis is how to realistically control virtual human response to unexpected perturbation. Many methods have been proposed to solve these problems [67–69]. Silei integrated physical simulation and motion data and designed a reactive human motion synthesis system which reacts accurately and simultaneously to the external forces under the premise of preserving the authenticity of motion data [70].

Many Chinese researchers work in the 3D human motion editing and synthesis area; examples include Luo et al. in video-based motion synthesis [7], motion retrieval [52, 71], key frame extraction from motion-captured data [48], group animation synthesis [72], and motion style synthesis [73]; Liu et al. in motion editing [74], motion retargeting [75], evaluation of motion data [76], and crowd evacuation [77]; Pan et al. in reactive motion synthesis [78]; Wei-Dong et al. in motion synthesis in martial arts [79] and cartoon animation [80]; Chen et al. in human motion path editing [81] and key frame interpolation [82]; Shen et al. in motion compression [47] and graphics processing unit-based crowd simulation [83]; Zhang et al. in feature detection [84] and video background subtraction [85].

## Figures and Tables

**Table 1:** Comparison of motion synthesis methods.

Motion synthesis methods	Advantages	Disadvantages
Manual methods	(1) Have the largest control of the generated motion (2) Can be used to generate motion for animals besides human beings	(1) Laborious and time consuming and require the animator to have rich experience

Physics-based methods	(1) Significantly decrease the time of manual adjustment (2) Generate improved results in mechanical and strong regular motion (3) Guarantee the motion in accordance with the physical law (4) Can be used to generate motion for animals besides human beings	(1) Difficult to use when producing smooth and emotional movements, such as dancing and sneaking (2) Generate motion in accordance with the physical law but are not natural and real (3) Entail high computational complexity (4) Feature a physical controller that is difficult to construct

Video-based methods	(1) Require simple data acquisition (2) Entail low cost equipment	(1) Require a single background for acquisition (2) Characterized by poor reusability of synthetic motion data (3) Extract movement data from videos with lower accuracy than motion capture data

Motion capture data-driven methods	(1) Can generate realistic and smooth motion (2) Feature low computational complexity (3) Showcase improved reusability of synthetic motion data	(1) Entail high costs for motion capture equipment (2) Generally applied only to human motion generation

**Table 2:** Categories of motion editing methods.

Motion attributes	Problems	Related work
Motion defect	Remove footskate after motion editing	[86]

Motion constraints	Motion editing algorithm based on a specific motion modifies demand (constraint set form)	[21, 87]

Motion path	Adjust the motion path	[81, 88]

Skeleton structure	Apply motion to a different structure of the skeleton (the same topology or not)	[75, 89–93]

Multilevel motion details	Process the motion data as signal using signal processing technology to edit the motion at different levels of details	[94, 95]

Physical properties	Combine dynamic constraints with energy law to edit the motion	[96]

Motion emotion	Apply the extracted emotion and mood to another motion	[97]
